# Using Inertial Measurement Unit Sensor Single Axis Rotation Angles for Knee and Hip Flexion Angle Calculations During Gait

**DOI:** 10.1109/JTEHM.2022.3226153

**Published:** 2022-12-01

**Authors:** Nuno Oliveira, Joonsun Park, Peter Barrance

**Affiliations:** School of Kinesiology and NutritionThe University of Southern Mississippi 5104 Hattiesburg MS 39402 USA; Department of Kinesiology and Health ScienceUtah State University 4606 Logan UT 84322 USA; Center for Mobility and Rehabilitation Engineering ResearchKessler Foundation 158368 West Orange NJ 07052 USA; Children’s Specialized Hospital Research Center New Brunswick NJ 08901 USA; Department of Physical Medicine and RehabilitationRutgers New Jersey Medical School 12286 Newark NJ 07103 USA

**Keywords:** Gait, hip, knee, inertial measurement units, validation

## Abstract

Background: Hip and knee flexion joint motions are frequently examined in clinical practice using camera based motion capture (CBMC) systems; however, these systems require elaborate setups and dedicated space. Inertial measurement unit (IMU) based systems avoid these disadvantages but require validation before widespread adoption. Moreover, it is important for clinical practice to determine the stability of these systems for prolonged evaluation periods. The purpose of this study was to assess the validity of a three-sensor inertial measurement unit system for calculating hip and knee flexion angles during gait by comparing with a gold standard CBMC system. Validity was also examined before and after a treadmill walking session. Methods: Twenty healthy participants were tested. Twenty seconds of gait at preferred walking speed were analyzed before and after thirty-two minutes of treadmill walking using previously validated CBMC methods and with a custom IMU model. Measurement validity for the IMU system was evaluated using Bland & Altman 95 percent limits of agreement, linear regression, mean absolute error and root mean square error. The effects of a measurement zeroing calibration strategy were also investigated. Results: Strong measurement agreement was observed for both hip and knee flexion angles, although overall agreement for the hip exceeded that for the knee. Linear regressions between the datasets for each participant illustrated strong (> 0.94) relationships between IMU and CBMC measurements. More significant changes between timepoints were observed for the knee than for the hip. Error values were generally reduced when zeroing calibration was implemented. Conclusion: The IMU system presented in this study is a convenient and accessible technique to measure joint angles. The protocol described in the current study can be easily applied in the clinical setting for evaluation of clinical populations. Additional development work on sensor placement and calibration methods may further increase the accuracy of such methods. Clinical translation statement: The IMU system presented in this study is a convenient and accessible technique to measure joint angles. Additional developmentwork on sensor placement and calibration methods may further increase the accuracy of such methods.

## I. Introduction

HIP and knee flexion are the joint motions with the largest ranges of motion during gait [1]. These joints can undergo large deviations from a healthy gait pattern in individuals with conditions that affect gait function. Large hip and knee flexion deviations have been reported for individuals with cerebral palsy [2], [3], stroke [4], traumatic brain injury [5], and multiple sclerosis [6]. Improving gait function in these populations typically involves long periods of gait retraining and assessment. Therefore, it is important to have tools that can evaluate and assess hip and knee flexion gait patterns during the rehabilitation process. Camera based motion capture (CBMC) systems are often considered the gold standard reference technology for kinematic measurements due to their proven and consolidated high accuracy [7], [8]. However, they have several characteristics that might limit their application in the clinical setting; for instance, they have time-consuming setup and calibration procedures and are limited to a motion capture volume within a designated zone. This might significantly limit the ability to regularly evaluate baseline kinematics and measure kinematic changes during rehabilitation.

Recent technological advancements have introduced alternative methods for motion capture and kinematic analysis of gait. Inertial Measurements Units (IMUs) consist of small sensors that are placed on body segments and measure changes in orientation and acceleration. This provides large flexibility in the development of systems for gait analysis by allowing many possible sensor configurations. IMU systems developed for gait analysis address the aforementioned limitations of CBMC systems, having easier and quicker setup procedures and not being limited to pre-calibrated spaces. Previous studies have examined the accuracy and precision of IMU system data during hip and knee angle measurements [9], [10], [11]. These studies reported consistent levels of good to excellent validity for knee and hip sagittal plane angles during different walking conditions. However, most studies tested IMU systems that have proprietary sensor configurations, models and software that can be expensive and might require an increased number of IMU sensors. In clinical applications these considerations may offset the advantages in cost and setup, motivating the investigation of simpler methods with low computational requirements and a low number of IMUs.

Limitations in precision resulting from sensor drift have been reported in IMU-based systems [12], [13]. Several strategies have been developed to correct for sensor drift, but these sensor systems still require periodic recalibration. Additionally, to avoid changes in sensor position and alignment, IMU sensors are typically tightly attached to the skin using tape or Velcro bands. However, the degradation of taping or displacement of the bands resulting from long periods of gait and sweat might impact the accuracy of the systems. For clinical applications, it is important to assess the stability of measurements for IMU based systems for gait evaluations across clinically relevant training or testing periods. Such assessments are limited in the current literature on validation for IMU systems, with most studies focusing on short term testing periods.

The purpose of this study was therefore to validate a three-sensor inertial measurement unit system that uses an easily implemented single axis rotation model for calculating hip and knee flexion angles during gait. To address the lack of prior information on long term measurement stability, knee and hip flexion angles measured using IMUs and a CBMC system were compared before and after a gait testing protocol of approximately 40 minutes duration.

## II. Methods

### A. Participants

This study included twenty healthy participants (7F, 13M; 20.7 ± 8.1 yrs.; 168.8 ± 13.5 cm; 63.5 ± 18.5 Kg). The following inclusion criteria were used: 10–40 years of age, able to understand spoken English, and able to walk without difficulty on a treadmill. Exclusion criteria included significant injury that interfered with the ability to walk, significant recent surgery, and known increased risk of stroke or heart attack. All research procedures were approved by the institutional human subject’s research review board.

### B. Instrumentation

Sixteen passive retro-reflective markers were attached to the participant for CBMC kinematic measurement. The retro-reflective markers were placed bilaterally at the ASIS, thigh, knee joint, shank segment, ankle, heel and toe according to the Plug-in Gait model [14]. Marker data were collected at 60 Hz with six infrared cameras (Qualisys, Göteborg, Sweden).

**FIGURE 1.**
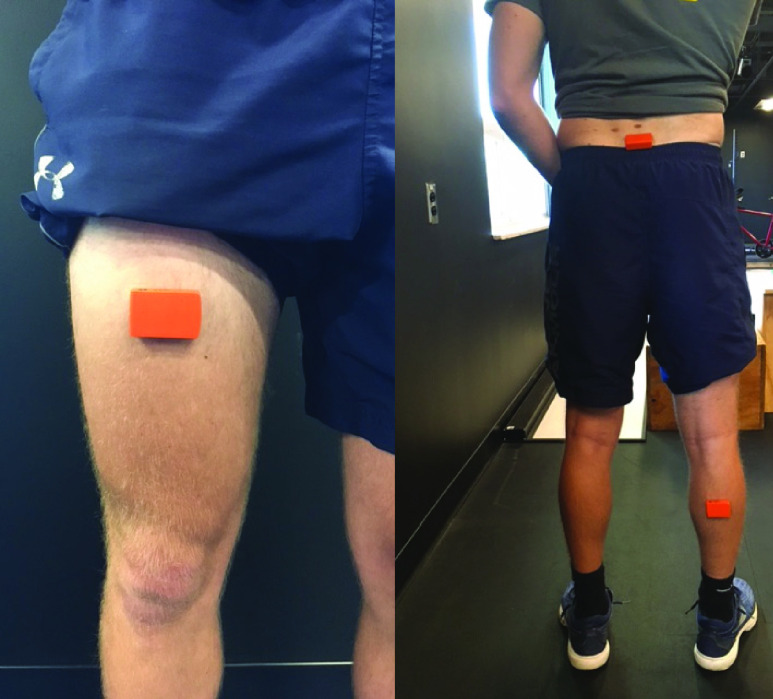
Motion sensors location and position for the IMU system.

Three IMU sensors (Xsens MTw, Enschede, The Netherlands) were placed on the participant while standing in the anatomical position. The sensor location was optimized based on preliminary development work: 1) the IMU sensors used to develop the current system (Xsens MTw, Enschede, The Netherlands) have optimal stability for rotations in the longitudinal axis (‘roll axis’) [15]. 2) The following sensor locations were determined to allow for the optimal alignment of the sensors along the longitudinal axis (‘roll axis’) while minimizing motion from soft tissue during walking. 3) We tried to define landmarks and procedures for the sensor locations that could be easily implemented in the clinical setting. Therefore, the following locations were established. One sensor was placed on the lower back over the posterior aspect of the sacrum, and the remaining sensors were placed on the right thigh and shank (Fig.1). The thigh sensor was placed on the anterior portion of the upper leg at half the distance from the anterior superior iliac spine to the superior part of the patella. The shank sensor was placed along the midline of the posterior portion of the lower leg at half the distance of tibia. The sensor alignment was horizontally exercised and placed to maintain the longitudinal axis of rotation (‘roll’) of the sensors parallel to one another [16]. Knee flexion angle was calculated as the difference between the thigh and shank sensors’ rotation about the sensor’s longitudinal axis (3), and hip flexion angle was calculated as the difference between the sacrum and thigh sensors’ rotation about the sensor’s longitudinal axis (4). Sensor data were collected at 60 Hz.

\begin{align*} Knee~Flexion=&{IMU}_{thigh}-{IMU}_{shank} \tag{1}\\ Hip~Flexion=&{IMU}_{sacrum}-{IMU}_{thigh}\tag{2}\end{align*}

Both retro-reflective markers and MTw motion sensors were securely attached using a double-sided adhesive tape. The MTw motion sensors at thigh and shank segments was wrapped in elastic wrap one more time then secured in place using athletic tape to prevent a sensor from moving from its original place.

### C. Testing and Protocol

The session started with familiarization to walking on the treadmill while selecting the preferred self-selected speed that was used for all testing (1.8 ± 0.3 m/s; range: 1.2 – 2.4 m/s). For each participant, a calibration trial was performed before the test. During the calibration, the participant was instructed to stand in the anatomical position for 10 seconds. Data was recorded from two trials in which participants walked on the treadmill at their preferred speed for 1 minute. For each trial, the second 20-second period of data was analyzed. Between the two trials, participants were involved in another study that consisted of walking on a treadmill while receiving visual feedback on their hip and knee flexion angles and performing appropriate modifications to their gait [17]. During this protocol, participants walked on the treadmill for four bouts of 8 minutes alternated with 3 minutes of rest, for a total testing time of 41 minutes.

### D. Output Measures

Raw motion analysis data were digitized in Qualisys Track Manager (v.2020.2, Qualisys, Göteborg, Sweden) and processed in Visual 3D (C-motion, Inc. Germantown, USA). CBMC knee flexion and hip flexion were calculated without (KF, HF) and with zeroing (KFcor, HFcor). Zeroing was performed by subtracting the respective average hip and knee flexion recorded during the static calibration trial from KF and HF.

For the motion sensors, knee and hip flexion angle calculations and recording was done using MATLAB (MathWorks Inc., Natick, MA). Knee flexion (KFIMU) was calculated as the difference in the orientation about the longitudinal axis of rotation (‘roll’) between the thigh sensor and the shank sensor. Hip flexion (HFIMU) was calculated as the difference between the roll of the thigh sensor and the pelvis sensor. The use of heading readings which derive from the magnetometer measurements and are susceptible to error magnetic field disturbances is avoided. Both KFIMU and HFIMU were zeroed by subtracting the respective offset recorded during the calibration step.

IMU and camera based signals were aligned in time by the optimization of a variable time offset using the cross correlation function in MATLAB.

### E. Statistical Analysis

A linear regression was used to determine the linear strength of relationship between the IMU and CBMC signals. The coefficient of determination ( $r^{2}$) indicated how much variance is shared between the IMU and CBMC. The coefficients $m$ (‘slope’) and $b$ (‘intercept’) were calculated to describe the relationship between IMU and CBMC. The mean absolute error (MAE) and the root mean square error (RMSE) were calculated to determine the average model prediction error in degrees. Bland & Altman 95 percent limits of agreement [18] were used to determine the agreement between the measurements from the motion sensors and the CBMC system, and to visualize systematic errors between the two methods. The mean of the differences ( $\text{M}_{\mathrm {dif}}$) or ‘bias’ was calculated as the mean of the differences between the IMU measurements (KF_IMU_, ${\mathrm {HF}}_{\mathrm {IMU}}$), and the CBMC measurements without (KF, HF) and with (KF_cor_, ${\mathrm {HF}}_{\mathrm {cor}}$) correction across all observations. The repeatability coefficient (RPC) was calculated as:

\begin{equation*} RPC=1.96\times Sd\tag{3}\end{equation*}

where Sd is the standard deviation of the differences between the IMU measurements (KFIMU, HFIMU), and CBMC measurements (KF, HF) across all observations. The upper (ULA) and lower (LLA) limits of agreement were calculated as:

\begin{equation*} LA=M_{dif}\pm RPC\tag{4}\end{equation*}

Paired sample t-tests were performed to determine differences between T1 and T2 for Mdif, RPC, Upper LA, Lower LA, r2, m, b, MAE, and RMSE. Statistical significance was set to $\alpha =0.05$.

## III. Results

**FIGURE 2.**
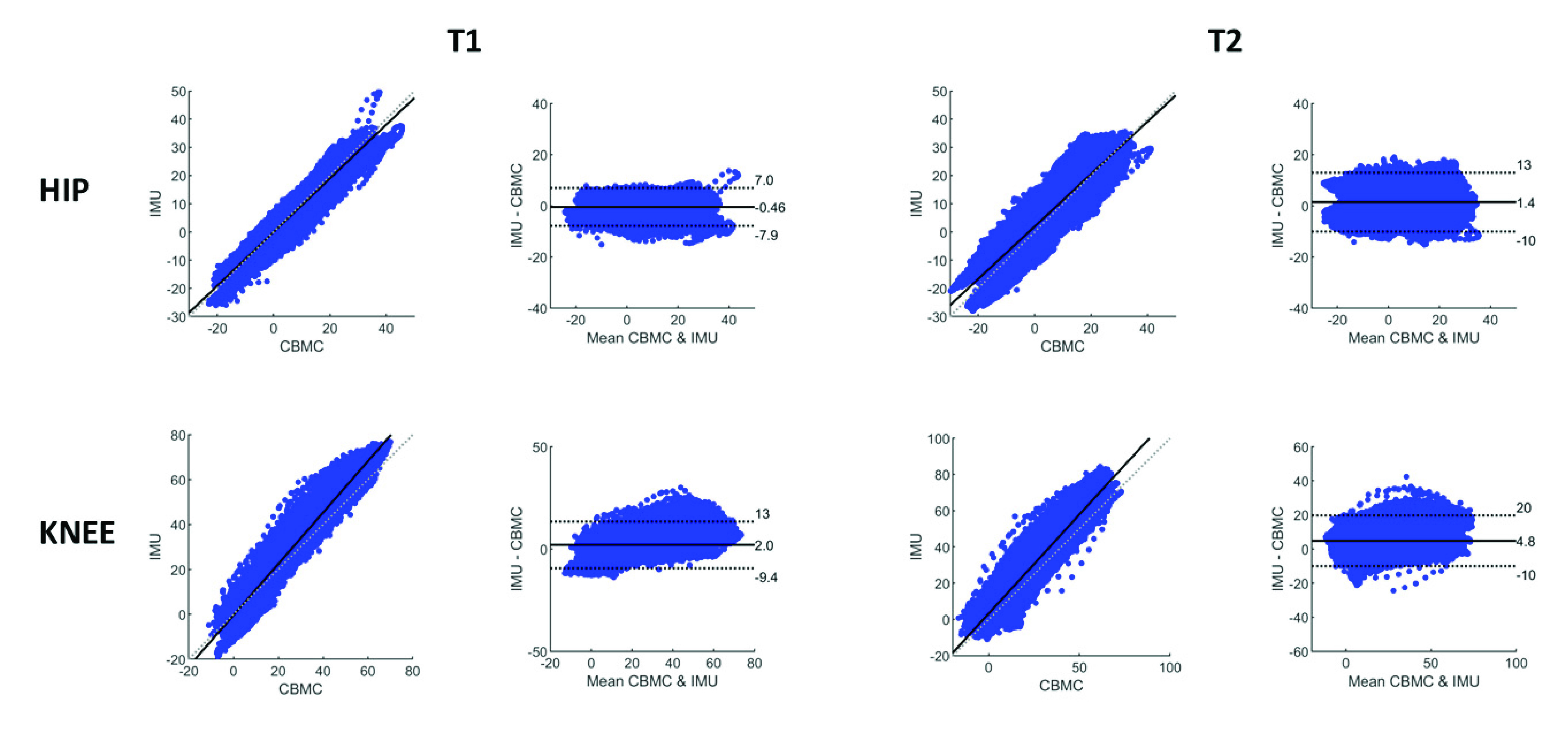
Correlation and Bland & Altman plots across all participants (24,000 data points) for hip and knee at time points T1 and T2.

Table 1 presents the results of the Bland & Altman analyses for corrected and uncorrected CBMC angles at the T1 and T2 time points (Fig. 2). For the uncorrected CBMC angles a stronger agreement for the hip than the knee was observed, with lower bias ( $\text{M}_{\mathrm {dif}}$) and RPC values. Bias was greatly reduced for knee joint angles when zero correction was implemented.

**TABLE 1:** Average Mean of the Differences ( $\text{M}_{\mathrm{dif}}$), Coefficient of Repeatability (RPC), Upper Limit of Agreement (Upper LA), Lower Limit of Agreement (Lower LA), Linear Regression Analysis (r^2^, m, b), Mean Absolute Error (MAE), and Root Mean Square Error (RMSE) Across Participants for Time Point One (T1) and Two (T2). Shaded Area Indicates That Values From Comparisons are the Same Between Raw and Zeroed Values

		$\text{M}_{\mathrm {dif}}$ (°)	RPC	Upper LA (°)	Lower LA (°)	r^2^	m	b (°)	MAE (°)	RMSE (°)
KF_IMU_ vs KF	T1	4.9 ± 6.1 a	9.1 ± 2.8	14.1 ± 6.5 a	−4.2 ± 7.0	0.97 ± 0.02	0.84 ± 0.06	−2.0 ± 5.9 a	7.4 ± 3.3 a	8.6 ± 3.3^a^
	T2	7.7 ± 6.2 a	9.3 ± 2.2	17.0 ± 6.9 a	−1.6 ± 6.4	0.97 ± 0.02	0.85 ± 0.06	−4.6 ± 6.6 a	9.3 ± 3.5 a	10.4 ± 3.5 a
HF_IMU_ vs HF	T1	−0.70 ± 6.8	4.1 ± 1.1 a	3.4 ± 7.0	−4.7 ± 6.8	0.98 ± 0.01 a	0.97 ± 0.06 a	0.90 ± 6.7	5.9 ± 3.1	6.3 ± 3.0
	T2	1.2 ± 6.9	4.7 ± 1.1 a	6.0 ± 7.0	−3.5 ± 6.5	0.97 ± 0.01 a	0.94 ± 0.06 a	−0.69 ± 6.5	5.7 ± 3.7	6.1 ± 3.6
KF_IMU_ vs KF_cor_	T1	2.0 ± 3.2 a		11.2 ± 4.0 a	−7.1 ± 4.5			0.94 ± 3.3 a	4.7 ± 1.3 a	6.0 ± 1.5 a
	T2	4.8 ± 5.9 a		14.1 ± 6.8 a	−4.5 ± 5.7			−1.6 ± 5.5 a	7.0 ± 3.5 a	8.2 ± 3.5 a
HF_IMU_ vs HF_cor_	T1	−0.45 ± 3.2		3.6 ± 3.2	−4.5 ± 3.6			0.80 ± 2.6	3.0 ± 1.6 a	3.5 ± 1.6 a
	T2	1.4 ± 5.4		6.2 ± 5.9	−3.3 ± 5.2			−0.90 ± 4.3	4.9 ± 2.8 a	5.4 ± 2.8 a

^a^ Indicates statistically significant differences (p < 0.05) between T1 and T2.

Linear regressions between the IMU and CMBC datasets for each participant yielded mean coefficients of determination between 0.94 and 0.98 (strong) [19], [20]. MAE and RMSE were larger for the knee compared to the hip, with both joints showing reduced MAE and RMSE when the zero correction was implemented.

Comparison of results between timepoints revealed differences over time for both joints (Table 1), with the following data reporting mean T1-T2 differences for all significant effects. Increased $\text{M}_{\mathrm {dif}}$ (KF: 2.8 ± 5.1°; KF_cor_: 2.8 ± 5.1°) and Upper LA (KF: 2.9 ± 5.1°; KF_cor_: 2.9 ± 5.1°) were observed for the knee, whereas hip RPC increased 0.6 ± 0.8 from T1 to T2. Reduced r^2^ (0.01 ± 0.01) and $m$ coefficient (0.03 ± 0.05) were observed for the hip (HF), while the b coefficient for the knee (KF and ${\mathrm {KF}}_{\mathrm {cor}}$) decreased from T1 to T2 (KF: −2.6 ± 4.7°; KF_cor_: −2.5 ± 4.7°). KF, KF_cor_ and HF_cor_ MAE increased (KF: 1.9 ± 2.9°; KF_cor_: 2.3 ± 3.0°; HF_cor_: 1.9 ± 5.0°), while KF, KF_cor_ and HF_cor_ RMSE (KF: 1.8 ± 2.8°; KF_cor_: 2.2 ± 2.8°; HF_cor_: 1.9 ± 2.7°) also increased.

## IV. Discussion

This study reports the validity of an easily implemented IMU based sensor measurement system for knee and hip flexion during gait by comparison with a standard camera based motion capture system. Measurement stability was evaluated by comparing the results before and after a testing protocol lasting approximately 40 minutes.

**FIGURE 3.**
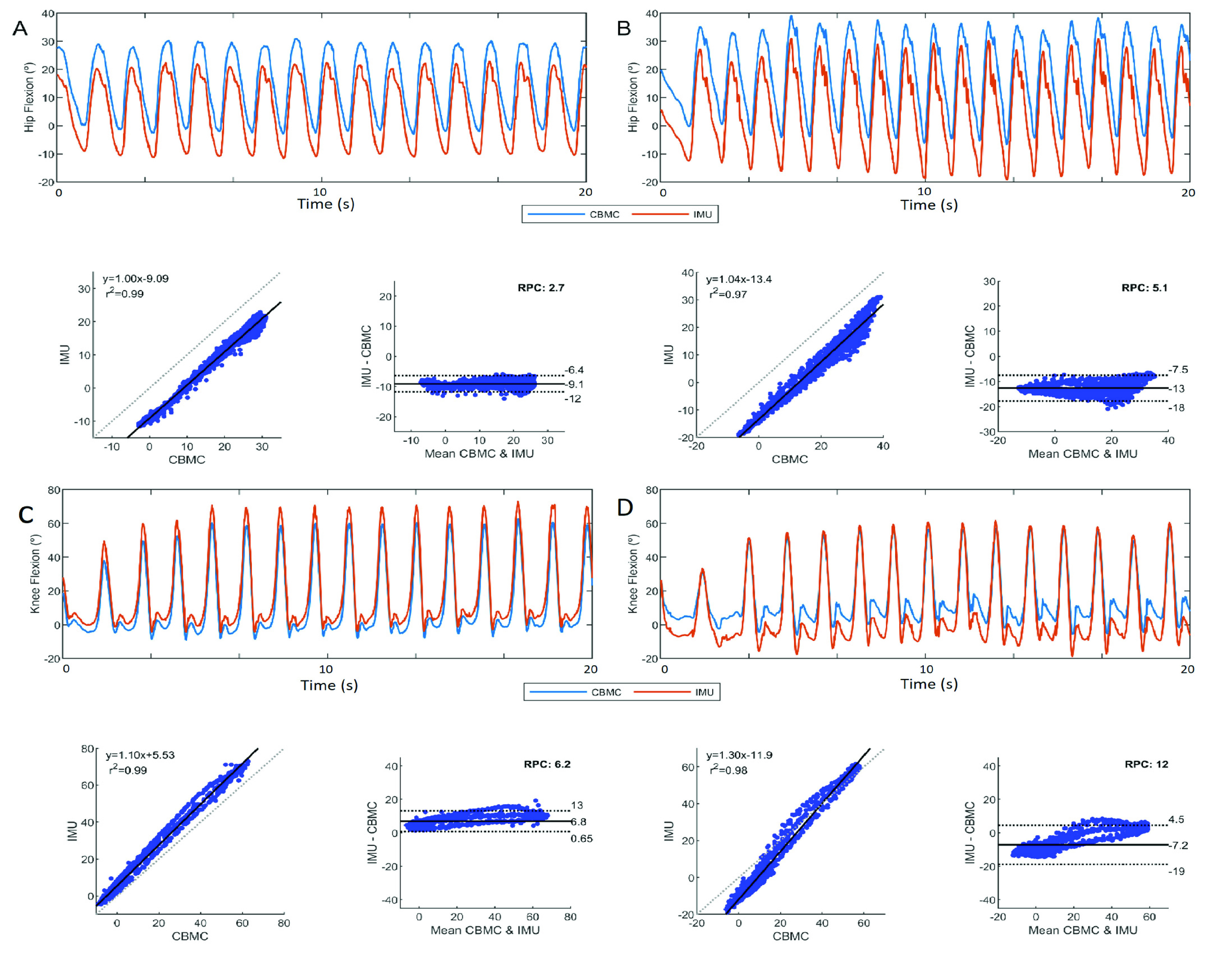
Joint angle patterns measured by the motion sensors (IMU) and camera system (CBMC), correlation (with coefficient of determination and linear equation) and Bland & Altman plots (with RPC, limits of agreement, and $\text {M}_{\text {dif}}$) of the participant with the lowest hip RPC (A), highest hip RPC (B), lowest knee RPC (C), and highest knee RPC (D). A and C occurred during T1, and B and D occurred during T2.

A stronger agreement for hip flexion angles than knee flexion angles was demonstrated in our study. $\text{M}_{\mathrm {dif}}$ and RPC for the hip joint were lower than the $\text{M}_{\mathrm {dif}}$ and RPC for the knee. $\text{M}_{\mathrm {dif}}$ for both the hip and the knee were reduced for comparisons with KF_cor_ and HF_cor_. For the hip, increased RPC might be explained by increased variability in measurement during peak hip flexion (Fig. 3, A, B). For the knee, there was a tendency to shift bias during excursion of the knee angle (Fig. 3, C, D). We believe that these are caused by movement of the motion sensors relative to the overall limb segments. Motion sensors are placed on the skin over muscles that contract during gait. Muscle contractions might modify the morphology of the body segment, changing the relationship between the orientation of the sensor and the overall segment. In this study, while the HF measurements involve a sensor that is placed on a muscle (thigh) and a sensor that is placed on the skin over a bone (sacrum), KF calculation use two sensors that are placed on the skin over muscles. Additionally, the intensity of the contractions can also be affected by individual morphology (e.g. muscle volume and shape) and gait speed. Reduction of artifact might be further improved by refinement of sensor locations and attachment methods.

The average coefficient of determination ( $r^{2}$) for the hip and knee across participants and time points was 0.97. This indicates a strong relationship between the motion sensors and the camera system even after a relatively prolonged use of the systems. The values for $r^{2}$ reported in this study are similar to previous validation studies of the MVN Biomech (Xsens, Enschede, NL) system during gait [9], [10]. Although this system uses the same sensors used in this study, it calculates hip and knee flexion angles using a proprietary biomechanical model. The average $m$ coefficient for the hip was 0.96, whereas the average $m$ coefficient for the knee was 0.85, indicating a tendency in the motion sensors to overestimate knee flexion rates. The value for the knee is supported by Liu et al. [21], in which a similar overestimation for knee flexion rate was reported when comparing the same IMU system to angles measured using a three camera motion capture system (Optitrack Trio, NaturalPoint, Inc., Corvallis, OR) in a single pediatric participant with hemiplegic cerebral palsy.

Average $b$ coefficients are close to zero for both hip and knee measurements. When comparing KF_IMU_ and HF_IMU_ to KF and HF, standard deviations indicate large individual variability. This variability in individual offsets might be a limitation when using the motion sensors across individuals. However, when comparing KF_IMU_ and HF_IMU_ to KF_cor_ and HF_cor_, variability was reduced, suggesting the importance of techniques that correct individualized offsets during calibration (e.g. measuring knee flexion angle with a goniometer). This is also supported by the reported MAE and RMSE. Both joints reduced MAE and RMSE when compared to the zeroed KF and HF values (KF_cor_ and ${\mathrm {HF}}_{\mathrm {cor}}$). Techniques to calibrate IMU angle readings to external measures are especially important in clinical populations with functional limitations affecting the ability to adopt standardized calibration poses [21].

The effect of time (T1 to T2) on measures of agreement between the IMU and CBMC methods were also analyzed in this study. Of the two joints analyzed, more significant evidence in changes were observed in the knee, with MAE and RMSE for KF_IMU_ increasing between timepoints by 1.9 ± 2.9° and 1.8 ± 2.8° respectively. No changes in $r^{2}$ or the $m$ coefficient were detected for the knee. There was however a significant change in the $b$ parameter for KF_IMU_ of −2.6 ± 4.7°. Considered alongside changes in other parameters, this implies a change in calibration offset between the two time points, with stable proportional measurement response to joint movements. Quantitatively, the average change in $b$ is within the range of reliability error (between 2° and 5°) suggested to be regarded as reasonable, but perhaps requiring extra consideration in data interpretation [22]. Moreover, the standard deviation of 4.7° showed that the difference in calibration offset was quite variable across participants, with some subjects showing larger calibration changes across the time period. On the other hand, in the hip the increased RPC, r^2^, and $m$ coefficient, with a stable $b$ coefficient, suggested a change in the proportionality of the IMU system’s response to hip flexion- although the significant difference in m was of small magnitude (0.03 ± 0.05). Both issues are likely related to changes in the orientation of the sensors relative to the overall limb morphology over time. This might be caused by declining effectiveness of the double-sided tape that attached the sensor to the skin or loosening of the athletic tape with time due to sweat and movement. In interpreting these results it should be noted that participants in our study walked on the treadmill for 41 minutes, which is considerably longer than standard clinical evaluation or gait analysis in clinical populations. Nevertheless, strategies to improve the attachment and stabilization of the motion sensors should continue to be investigated.

The findings of this study support the use of our inertial measurement unit system for knee and hip flexion angle calculations to investigate gait changes in a variety of clinical populations, particularly when combined with appropriate measurement calibration. These values are smaller than typically reported clinical gait deviations for hip and knee flexion. For example, patients with Parkinson’s disease reported an average 12 degrees of hip flexion deviation during late stance phase (terminal stance and pre-swing phases) and an average 9 degrees of knee flexion deviation during mid swing phase [23]. Moreover, an average of 5.9 degrees of deviation for hip extension, and an average 17.4 degrees for the knee flexion have been observed during post-stroke gait [24]. Finally, typically reported gait deviations in individuals with cerebral palsy (crouch gait) involve at least 10 degrees of increased knee flexion [2], [25]. Methodological steps including calibration strategies that can optimize the application of the current system in specific clinical populations should also be investigated.

The current study investigated the validity of a three-motion sensor configuration to measure hip and knee flexion angles during gait. A strong relationship between the motion sensors and the CBMC was observed for hip and knee angles, while hip angles exhibited lower measurement bias as well as higher measurement stability over time than knee angles. When hip and knee flexion angles from the CBMC system were corrected with the calibration offset, MAE, RMSE, and $\text{M}_{\mathrm {dif}}$ were reduced. We should note that the current system was developed using commercially available IMU sensors (Xsens MTw) that provide the orientation of each sensor as an output that was used by our MATLAB program. Other commercially available IMU sensors might not provide this output directly and sensor orientation might have to be calculated from raw data. Nevertheless, the single axis rotation technique for calculating knee and hip flexion angles evaluated in this study should be feasible across different commercially available devices. Additional development work on sensor placement and calibration may further increase the accuracy of such methods.

## V. Conclusion

The current study investigated the validity of a three-motion sensor configuration to measure hip and knee flexion angles during gait. A strong relationship between the motion sensors and the CBMC was observed for hip and knee angles, while hip angles exhibited lower measurement bias as well as higher measurement stability over time than knee angles. When hip and knee flexion angles from the CBMC system were corrected with the calibration offset, MAE, RMSE, and $\text{M}_{\mathrm {dif}}$ were reduced. Additional development work on sensor placement and calibration may further increase the accuracy of such methods.
